# Transfer of lead from soil to pasture grass and milk near a metallurgical complex in the Peruvian Andes

**DOI:** 10.1093/tas/txab003

**Published:** 2021-01-13

**Authors:** Doris Chirinos-Peinado, Jorge Castro-Bedriñana, Edgar García-Olarte, Rolando Quispe-Ramos, Stephane Gordillo-Espinal

**Affiliations:** 1 Center for Research in Food and Nutritional Security, Universidad Nacional del Centro del Perú, Huancayo, Perú; 2 Faculty of Zootechnics, Universidad Nacional del Centro del Perú, Huancayo, Perú

**Keywords:** contaminated grass, contaminated milk, contaminated soils, dairy milk, heavy metals, smelting emissions

## Abstract

Milk quality is affected by the concentration of lead (Pb) in soil and pasture grasses used to raise cattle, especially in areas near mining-metallurgical complexes. In this study, the Pb content of soil and its transfer to grass and dairy milk in an area located to 20 km from the La Oroya Metallurgical Complex in Peru (altitude, >3,700 m s.a.l) was measured. Twenty soil samples (0–30 cm depth), 20 grass samples were collected, and 0.5 L of milk was obtained from 20 lactating cow in the communal cowshed. The Pb concentration (mg/kg) was quantified by flame atomic absorption spectrometry using a commercial Pb standard as quality control. The Pb average concentration in the soil, grass, and milk samples were 217.81 ± 39.48, 20.09 ± 2.83, and 0.58 ± 0.018 mg/kg (*P* < 0.01), respectively. The transfer factor (ratio of metal concentration) for Pb from soil to grass and from grass to milk was 0.095 and 0.031, respectively. The soil, grass, and milk samples all presented high Pb concentrations, with the milk samples containing 29-fold more Pb than the safety limit established by European regulations and were not suitable for human consumption or the manufacture of cheese, yogurt, and other derivatives. Our findings demonstrate that action to remediate these soils is critically needed.

## INTRODUCTION

Contamination of soils and plants with toxic metals such as lead (Pb) is a global concern (Zhang et al., 2019). Lead transfer to the food chain endangers food safety and human and animal health (Tong et al., 2000); and represents a significant risk factor to exposed populations (Rai et al., 2019; Safiur Rahman et al., 2019).

The importance of milk consumption is widely recognized and documented, especially in the pediatric population (Bishop MacDonald, 2010). Milk production and quality are affected by environmental conditions (Zhou et al., 2019) and dictate the concentration of competitiveness of the dairy chain (Ding et al., 2019). Raw milk is considered a product of high importance to public health, and milk quality and safety can be compromised by improper product handling, from production to consumption. Milk may become contaminated with biological or chemical hazards (Iqbal et al., 2016) that affect the quality and nutritional value of milk products, such as contamination with heavy metal from multiple sources (Jahed Khaniki, 2007; Patra et al., 2008; Tajkarimi et al., 2008).

Although mining is an important revenue-generating activity in Peru and represents the highest percentage of net exports (Loayza and Rigolini, 2016), mining and metallurgical activities generate fine particulate matter with heavy metals (Imperato et al., 2003).

This particulate matter can be carried over long distances by wind and deposited in areas contaminating water, soil, plants, animals, and humans. This entry into the food chain causes irreversible damage due to its long biological half-life, high toxicity, and high potential for bioaccumulation in the body (Imperato et al., 2003; Ogabiela et al., 2011; Sing et al., 2012; Barenys et al., 2014; Bortey-Sam et al., 2018; Antoniadis et al., 2019; Yu et al., 2019). There is a high concern regarding the dietary exposure to heavy metals (Barenys et al., 2014).

The La Oroya Metallurgical Complex is in Yauli Province (altitude, 3,740 m) in the Central Andes and has been operational since 1922 (Reif et al., 1989). La Oroya has long been known as one of the most polluted cities in the world (Olympio et al., 2017). No published studies that have measured metal concentrations in local soils, dust, drinking water, or heavy metal concentrations in the air; however, a study in La Oroya has found high concentrations of lead in blood samples from almost all children under 6 years old; high concentrations of cadmium, arsenic, mercury, antimony, and cesium (Fraser, 2006).

The smelting of non-ferrous metals has created a chronic public health problem in La Oroya, contaminated soils in the vicinity of the smelter, which recovers Cu, Pb, Zn, is a serious health problem (Reuer et al., 2012). However, there is no information on the dynamics of Pb contamination and bioaccumulation in dairy products from these areas.

The soil is a limited and fragile resource that must be protected against erosion and heavy metal contamination because soil damage can affect agrosilvopastoral systems (Zhang et al., 2020). Heavy metals are transferred from the soil to ecosystems and enter the food chain (Huang et al., 2019; Mehmood et al., 2019; Yan et al., 2019). In the soil, Pb is concentrated in grass roots (Nascimento et al., 2014) and its absorption and accumulation affect pasture yield (Alloway, 2013; Olayinka et al., 2017). Moreover, Pb also accumulates in the milk of grazing dairy cows, resulting in a serious public health concern (Ogabiela et al., 2011). Toxic metals have a long biological half-life and high bioaccumulation and transfer potential (Sing et al., 2012; Bortey-Sam et al., 2018; Antoniadis et al., 2019; Yu et al., 2019).

Lead exposure in humans affects hematogenesis, as well as the nervous, reproductive, cardiovascular, digestive, and urogenital systems. Furthermore, Pb toxicity delays intellectual development, decreases cognitive function (Tepanosyan et al., 2017; López-Rodríguez et al., 2017; Bortey-Sam et al., 2018), and causes metabolic disorders (Rai et al., 2019) and cancer (Lu et al., 2014, 2018; Yu et al., 2019). Because Pb has a biological half-life of more than 25 years and chemical structure like that of calcium, Pb is deposited in bones, from where it is quickly mobilized during specific physiological stages, including pregnancy and lactation (Li et al., 2018). Milk is highly susceptible to contamination by heavy metals (Skipin et al., 2016); therefore, the concentration and bioaccumulation of Pb in milk should be monitored so that adequate evidence-based control measures can be implemented.

In populations surrounding La Oroya, 85% of children had more than 10 ug/dL of Pb (Astete et al., 2009), the or being many agri-food products contaminated with this heavy metal that then reach the final consumers. Reuer et al. (2012) at locations between 1 and 26 km from the La Oroya semlting complex report elevated Pb values in soil, indoor dust and drinking water, leading to chronic public health problems. Castro et al. (2013, 2016) report high concentrations of Pb in maternal blood, placenta, and umbilical cord blood with negative effects on gestational age, weight, length, and Hb in newborns.

To the best of our knowledge, no studies have determined Pb concentrations in milk produced in highly contaminated areas in the Peruvian Andes. In this study, we measured the Pb concentration in the soil and its transfer to pasture grasses and dairy milk in an area contaminated with emissions from mining and metallurgical activities.

## MATERIAL AND METHODS

### Study Site and Period

The present study was conducted from April to May 2018 in a rural community comprising approximately 315 families in the district of Paccha, Yauli Province, in the region of Junín, Peru (altitude, 3,745 m a.s.l. 11°31′03″S, 75°53′58″W). The study site is located approximately 20 km from the La Oroya Metallurgical Complex; cattle are raised there, and milk and its derivatives are sold in the markets of La Oroya, a city with more than 33,000 inhabitants. The lands are used primarily for livestock production, including approximately 1,500 sheep, 150 cattle, and 50 alpacas. The community has a dairy shed and a dairy processing plant with basic equipment, and milk production varies between 200 and 350 L/day, depending on the season and number of lactating animals. The area includes 11 ha of natural grassland composed of *Festuca dolichophylla*, *Piptochaetium featherstonei*, *Bromus catharticus*, *B. lanatus*, and *Calamagrostis heterophylla*.

The metallurgical complex was owned by an American company from 1922 to 1974 and was nationalized in 1974. In 1997, the complex was sold to Doe Run Company, a subsidiary of the American Renco Group16, and was fully operational until 2008. The metal smelting operations were interrupted in 2009, and since then, the complex has only been functioning partially.

### Sampling of Soil, Grass, and Milk

Twenty sampling points were selected in the natural grasses of the study site, from which 20, 1 kg surface soil samples (0–30 cm depth) and corresponding 20 grass samples were collected. The samples were dried, homogenized, transferred to Ziploc bags, labeled, and transported to the laboratory; soil and grass samples were collected from the same experimental areas to ensure a corresponding Pb content between samples (Hao et al., 2009). The grass was cut flush using clean, stainless-steel scissors, and then transferred to paper bags, labeled, and transported to the laboratory for pretreatment and analysis.

Milk samples were collected from 20 crossbred (Criollo × Brown Swiss) cows according to the protocol of the Peruvian Technical Standard N° 2020.115 (NTP, 2013). From each cow, 0.5 L of milk was collected in wide-mouth, opaque polyethylene bottles previously washed with deionized water and transported to the laboratory under cold storage.

### Analysis of Pb Samples

#### Soil and grass.

EPA Method 3050B (SW-846): Acid digestion of sediments, sludges, and soils was followed. This method involves the digestion of soil samples and analysis by flame atomic absorption spectrometry (FAAS). For digestion, 1 g of dried sample was treated with nitric acid (HNO_3_) and hydrogen peroxide. For FAAS analysis, hydrochloric acid (HCl) was added to the digest and the sample was refluxed to increase metal solubility. The digest was filtered, and the filter paper containing the particulate matter was rinsed first with hot HCl and then boiling water. The material was returned to the digestion flask, refluxed with HCl, filtered again, and diluted to a final volume of 100 mL. Sample quality was assessed using a Pb standard (Sigma-Aldrich, USA) (986 ± 4 mg/kg). The reagents used were HNO_3_ (1:1), HNO_3_ [cc], HCl [cc], and 30% hydrogen peroxide. The material was homogenized and ground and foreign particles were removed. The samples were sieved through a 2-mm sieve, dried in an oven at 30–35 °C for 4 h, transferred to 250-mL beakers, treated with 10 mL of HNO_3_ (1:1), and heated at 95 ± 5 °C for 15 min. Then, 5 mL of HNO_3_ [cc] was added until the reaction was complete and the samples were concentrated to 5 mL. Two milliliters of water and 3 mL of 30% hydrogen peroxide (until minimal effervescence) were added to each sample, following which the reaction was concentrated to 5 mL, filtered, and transferred to 100-mL vials. Quality controls were applied and calibration curves were constructed. The material was analyzed by FAAS, and the results were reported as mg/kg.

The procedure for digestion of grass samples was like that for soil samples. Lead standards (Sigma-Aldrich) (160 ± 0.1 mg/kg) were used for quality control.

#### Milk.

AOAC Official Method 973.35 Lead in Evaporated Milk: Atomic Absorption Spectrophotometric Method (Latimer, 2016) was followed. A total of 50 g of the dried sample was digested with HNO_3_. For FAAS analysis, as an additional step to increase Pb solubility, the digest was filtered, and the filter containing the particulate matter was rinsed with boiling water. The digest was diluted to a final volume of 100 mL. A Pb standard (15 ± 0.04 mg/kg) was used for quality control. A total of 50 g of raw milk was weighed in 100-mL porcelain crucibles, dried in an oven at 100 °C until constant weight, and then ashed in a muffle furnace at 450 °C for 16 h. After cooling, the ash was bleached using 2 mL of 2 N HNO_3_, and the acidified samples were dried on a hot plate. The acid was evaporated and the samples ashed again in a muffle furnace at 450 °C for 1 h. Extraction was performed using 5 mL of 2 N HNO_3_ and 20 mL of 0.1 N HNO_3_. The samples were filtered using a Whatman No. 40 filter paper, stored in polypropylene tubes, and maintained under refrigeration. The Pb concentration was reported as mg/kg. All analyses were performed in duplicate.

### Lead Transfer from Soil to Grass and Milk

Since plants absorb and bioconcentrate a large part of the Pb present in the soil (Bech et al., 2012; Kumar et al., 2017; Rai et al., 2019), the determination of the transfer factor of heavy metals to the edible part of pastures in relation to the total soil content is an appropriate method to determine the capacity of plants to mobilize and capture heavy metals (Violante et al., 2010).

The soil-to-grass transfer factor (TF) for Pb was determined by dividing the Pb concentration in grass by the concentration in soil (Papaioannou et al., 2018). A similar method was used to determine the grass-to-milk and soil-to-milk TFs. Values for TF greater than 1 indicated the effective transfer of Pb from grass to milk.

### Statistical Analysis

Data analysis was performed using SPSS version 23 software. A one-sample *t*-test was used to assess whether the average Pb content in soil, grass, and milk exceeded the permissible limits. The maximum limits used in the tests in soil, grass, and milk samples were 70, 30, and 0.02 mg/kg, respectively. For statistical comparison between Pb concentrations in soil, grass, and milk samples One-way ANOVA was performed. A general procedure for linear regression models and analysis of variance were performed using SPSS software version 23 (IBM). Differences between means were assessed using Tukey’s test and *P*-values <0.05 were considered significant.

## RESULTS

### Lead Concentration in Soil, Grass, and Milk

Lead content was highest in the soil (*P* < 0.01), followed by grass and milk (Table 1).

**Table 1.** Lead concentrations in soil, grass, and milk samples

Parameter	Average	SD	Min.	Max.
Lead concentration in soil, mg/kg	217.81a	39.480	131.76	284.13
Lead concentration in grass, mg/kg	20.09b	2.830	14.55	23.88
Lead concentration in milk, mg/kg	0.58c	0.018	0.54	0.60

Average Pb concentrations in soil, grass, and milk, with different letters are statistically different at *P* < 0.01.

SD = standard deviation.

### Lead Transfer from Soil to Grass and Milk

The soil-to-grass TF for Pb was higher than that from grass to milk and soil to milk (Table 2).

**Table 2.** Descriptive statistics for the transfer factor (TF) for Pb from soil to grass and milk (*n* = 20)

Parameter	Average	SD	Min.	Max.
TF from soil to grass	0.0953	0.0228	0.0593	0.1460
TF from pasture to milk	0.0313	0.0088	0.0233	0.0639
TF from soil to milk	0.0027	0.0006	0.0021	0.0046

SD = standard deviation.

### Lead Concentration in Soil, Grass, and Milk According to Permissible Limits

The average Pb concentration in the soil at Paccha (217.81 mg/kg) was significantly higher (*P* < 0.01) than the maximum limit established by the Ministry of the Environment (MINAM, 2017) and the Canadian agricultural soil quality guidelines for environmental and human health protection (CCME, 1999) (70 mg/kg). This demonstrates that a large quantity of Pb has accumulated in the soil over decades, and this contaminant will remain in the food chain for many years.

In this study, the average Pb content in pasture grasses (20.09 mg/kg) was higher than the international reference value reported by Kabata and Pendias (2004), in which normal concentrations ranged from 5 to 10 mg/kg, tolerable concentrations varied between 0.05 and 10 mg/kg, and toxic concentrations ranged from 30 to 300 mg/kg.

Lead content in all the milk samples was significantly higher (*P* < 0.01) than the acceptable limits. The average Pb content in milk produced in study zone (0.58 mg/kg) was statistically higher than the maximum limit allowed for raw milk in Europe, 0.02 mg/kg (EC Regulation, 2006), demonstrating that the milk produced under the environmental conditions evaluated in this study was not suitable for either human or animal consumption.

**Figure 1.**
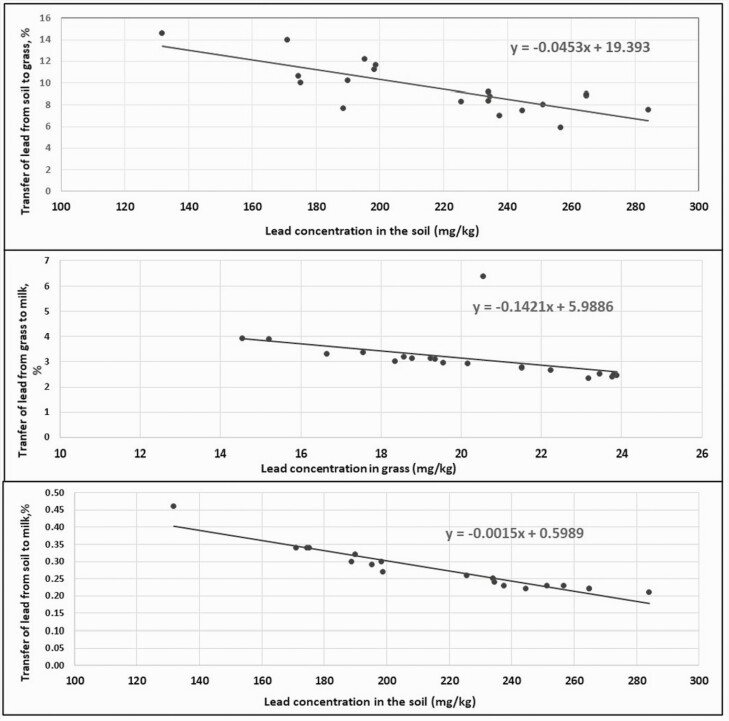
Regression lines of Pb concentration in the soil and grass, and Pb transfer percentage from soil to grass, grass to milk and soil to milk.

Figure 1 shows the regression lines of Pb concentration in the soil and the percentage of Pb transfer from soil to grass; Pb concentration in grass and the percentage of Pb transfer from grass to milk; and Pb concentration in the soil and the percentage of Pb transfer from soil to milk. The correlation coefficients varied between −0.45 and −0.94. The TF from soil to grass, grass to milk, and soil to milk decreased as the Pb concentration increased.

## DISCUSSION

Based on the national regulations in Peru, the Pb concentration in the study soils, where pastures are used to raise cattle, sheep, and camels, was 3.1-fold higher than the upper limit (70 mg/kg) established by the Ministry of the Environment (MINAM, 2017), 3.6-fold higher than the limit allowed by the Finnish Ministry of the Environment (2007), and 4.3-fold higher than the maximum limit reported by Kabata-Pendias and Pendias (2001). The Pb concentration in our study varied between 100 and 400 mg/kg, which is considered toxic (Ross, 1994).

The Pb content in pasture grasses was like that reported in Pakistan (Khan et al., 2015), but lower than that in Nigeria (Ogundiran et al., 2012) at sites containing Pb slags (425 ± 79.0 mg/kg). In contrast, Pb concentrations in our study were 10-fold higher than the limits tolerated in Germany (2 mg/kg) (KTBL, 2004; cited by Li et al., 2005) and 20-fold higher than the maximum concentration allowed in the United Kingdom (1 mg/kg) (Nicholson et al., 1999). The Pb concentration was 6.7-fold higher than the acceptable concentration for vegetables (0.05–3.0 mg/kg) (Grimshaw et al., 1989, cited by Tokalioglu et al., 2000) and higher than that found in New Zealand (Martin et al., 2017), Wisconsin (United States) (Li et al., 2005), leading to Pb accumulation in the food chain, especially in agricultural systems (Shah et al., 2010).

The average Pb concentration in milk produced (0.58 mg/kg) was above the upper limit (0.02 mg/kg) established by Codex Alimentarius (2007) and the European Union and the EC Regulation, 2006 and consumption of this milk or its derivatives could result in chronic intoxication (Sing et al., 2012; Assi et al., 2016). These values were higher than those reported in Nigeria near sites containing Pb slags (0.35 ± 0.14 mg/L) (Ogundiran et al., 2012). Ogabiela et al. (2011) evaluated cows grazing in the Challawa industrial area and non-industrialized areas in the state of Zaria Kaduna, Nigeria, and found that the average concentration of Pb in raw milk was 0.55 ± 0.32 and 0.71 ± 0.35 mg/kg, respectively, values that were above the maximum limits recommended by the World Health Organization.

In Multan, Pakistan, the Pb content in raw and commercial milk was found to be high (0.048–0.418 mg/L), which was attributed to industrial and agricultural mismanagement and inadequate sanitary measures during animal feeding and milking (Akhtar et al., 2015). In China, the average Pb content in raw milk was1.75 μg/L (Zhou et al., 2018); in others studies, the ranges of Pb in milk samples were 0.03–10.46 μg/L (Zhou et al., 2019). Data showed that the highest concentrations of Pb (60 mg/L) were noticed in raw cow milk collected in area consists of granites and granite gneisses in India (Boudebbouz et al., 2021).

When analyzing the minimum and maximum values of Pb content in the soil–plant–milk, it is observed that the range is much narrower in plants and then even narrower in milk, an aspect linked to mineral homeostasis in plants and animals, which should be investigated.

La Oroya is one of the most polluted cities in the world (Olympio et al., 2017). In the present study, milk contamination with Pb resulted from the accumulation of fine particulate matter emissions from the La Oroya Metallurgical Complex during almost 100 years of operation. Further studies are therefore necessary to identify areas at increased risk of heavy metal contamination based on the recommendations of Akhtar et al. (2015), as well as develop evidence-based guidelines establishing acceptable concentrations of heavy metals in soil, plants, and milk products in Peru.

The milk produced in this area, due to its high Pb content, could cause toxicity due to its consumption by the local population, especially children, who are the most vulnerable (Astete et al., 2009; Reuer et al., 2012; Castro et al., 2013, 2016). In children, the common route of exposure is the ingestion of lead-contaminated products; however, environmental and health impacts have not been established for the region (CDC, 2005).

In Peru, the milk per capita consumption per year is 87 kg, and if it contains 0.58 mg/kg of Pb, it would result in a daily intake of 0.14 mg of Pb (0.98 mg/week), which would represent 65% of the risk value for a 60 kg person (EFSA, 2010).

The soil-to-grass TF for Pb (0.0953 ± 0.0280 mg/kg) recorded in this study agrees with the results of Li et al. (2005), who reported a low soil-to-grass TF for Pb (approximately 0.1).

Linear regression and Pearson correlation analyses indicated that the TF for Pb to grass and milk decreased with increasing Pb concentrations in the soil (*P* < 0.01), which is consistent with previous results showing that the TF was dependent on multiple factors, as the capacity of plants to mobilize and capture heavy metals (Violante et al., 2010). This indicated that Pb transfer to grass and milk decreased as the concentration of Pb in the soil increased (Nascimento et al., 2014). Wang et al. (2002) observed that bioconcentration usually decreased with increasing Pb concentrations in the soil, this indicates that bioconcentration factors might be higher in uncontaminated soils than in contaminated soils.

## CONCLUSIONS

In the central Andes of Peru, where there is sustained metallurgical mining activity, Pb concentrations were significantly higher in the soil than in pasture grasses or milk, and Pb concentrations in the soil and milk exceeded those recommended by national and international standards. This indicates that milk produced under these conditions is unsuitable for human consumption, as well as for the manufacture of cheese, yogurt, and other dairy products. The data obtained in the evaluated ecosystem will be useful for designing and implementing strategies to reduce the adverse effects on human health associated with the consumption of contaminated milk and will also aid in establishing guidelines for acceptable concentrations of heavy metals in soil, plants, and milk in Peru.

Studies on Pb contamination in other dairy products, beef and sheep meat and other agricultural foods, should be continued, as they constitute a major environmental and public health problem that must be escalated.
